# Ursolic Acid Increases Glucose Uptake through the PI3K Signaling Pathway in Adipocytes

**DOI:** 10.1371/journal.pone.0110711

**Published:** 2014-10-20

**Authors:** Yonghan He, Wen Li, Ying Li, Shuocheng Zhang, Yanwen Wang, Changhao Sun

**Affiliations:** 1 Department of Nutrition and Food Hygiene, Public Health College, Harbin Medical University, Harbin, Heilongjiang, People’s Republic of China; 2 Aquatic and Crop Resource Development, Life Sciences Branch, National Research Council Canada, Charlottetown, Prince Edward Island, Canada; 3 State Key Laboratory of Genetic Resources and Evolution, Kunming Institute of Zoology, Chinese Academy of Sciences, Kunming, Yunnan, People’s Republic of China; 4 Department of Endocrinology, Third People's Hospital of Yunnan Province, Kunming, Yunnan, People’s Republic of China; 5 Department of Biomedical Sciences, University of Prince Edward Island, Charlottetown, Prince Edward Island, Canada; China Medical University, Taiwan

## Abstract

**Background:**

Ursolic acid (UA), a triterpenoid compound, is reported to have a glucose-lowering effect. However, the mechanisms are not fully understood. Adipose tissue is one of peripheral tissues that collectively control the circulating glucose levels.

**Objective:**

The objective of the present study was to determine the effect and further the mechanism of action of UA in adipocytes.

**Methods and Results:**

The 3T3-L1 preadipocytes were induced to differentiate and treated with different concentrations of UA. NBD-fluorescent glucose was used as the tracer to measure glucose uptake and Western blotting used to determine the expression and activity of proteins involved in glucose transport. It was found that 2.5, 5 and 10 µM of UA promoted glucose uptake in a dose-dependent manner (17%, 29% and 35%, respectively). 10 µM UA-induced glucose uptake with insulin stimulation was completely blocked by the phosphatidylinositol (PI) 3-kinase (PI3K) inhibitor wortmannin (1 µM), but not by SB203580 (10 µM), the inhibitor of mitogen-activated protein kinase (MAPK), or compound C (2.5 µM), the inhibitor of AMP-activated kinase (AMPK) inhibitor. Furthmore, the downstream protein activities of the PI3K pathway, phosphoinositide-dependent kinase (PDK) and phosphoinositide-dependent serine/threoninekinase (AKT) were increased by 10 µM of UA in the presence of insulin. Interestingly, the activity of AS160 and protein kinase C (PKC) and the expression of glucose transporter 4 (GLUT4) were stimulated by 10 µM of UA under either the basal or insulin-stimulated status. Moreover, the translocation of GLUT4 from cytoplasm to cell membrane was increased by UA but decreased when the PI3K inhibitor was applied.

**Conclusions:**

Our results suggest that UA stimulates glucose uptake in 3T3-L1 adipocytes through the PI3K pathway, providing important information regarding the mechanism of action of UA for its anti-diabetic effect.

## Introduction

The prevalence of diabetes has dramatically increased and results in a considerably higher rate of mortality worldwide. Insulin resistance has been considered as a well-known metabolic disorder of diabetes, which is closely related with serious complications such as cardiovascular and kidney diseases [1]. Insulin is critical in glucose homeostasis and stimulates the transport of blood glucose into the cells for metabolism in the peripheral tissues (such as muscle, fat tissue and liver) by regulating the expression and translocation of glucose transporters [2], [3]. When insulin resistance occurs, the insulin-mediated glucose uptake is impaired, leading to reduced glucose uptake into muscle, adipose or liver cells and consequently the elevation of blood glucose concentration. Accordingly, agents with an ability of stimulating glucose uptake in these tissues are used to improve or treat insulin resistance and diabetes.

Although some effective therapeutic drugs have been developed and used for many years to treat diabetes, most of these drugs produce undesirable or severe side effects, such as induction of fat accumulation [4], inhibition of hepatic regeneration [5], and causing osteoporosis [6]. Therefore, there is a need to develop new anti-diabetic products, especially through an approach of stimulating glucose uptake and utilization in peripheral tissues without causing obvious side effects. In this regard, natural products have provided a new avenue and been considered having great potential.

Ursolic acid (UA) is a natural pentacyclic triterpenoid and present in many different plants, fruits and herbs. Accumulating evidence has shown that UA possesses multiple nutritional and pharmacological functions [7], [8]. Recent studies have revealed that UA decreases weight gain and abdominal fat mass in mice fed a high-fat diet [9], [10]. Further studies have demonstrated that UA reduces adiposity by enhancing lipolysis [11], [12] and inhibiting adipogenesis [13]. It is well established that central obesity is closely related to insulin resistance and diabetes [14]. We hypothesized that UA might be beneficial to insulin resistant or diabetic patients partially through regulating fat and glucose metabolism. Indeed, emerging evidence demonstrates that UA is able to lower blood glucose and improve insulin resistance and diabetes [15]–[19]. Although several mechanisms have been reported, such as promoting the glucose uptake and utilization in muscle cells [20], [21], increasing liver glycogen synthesis and deposition [22], and increasing pancreatic β-cell function [16], it is not clear whether and how UA modulates glucose uptake and metabolism in adipose tissue, which plays an important role in glucose homeostasis through taking up glucose when the circulating glucose level is elevated [23]. Accordingly, the present study was conducted to determine the effect of UA on glucose uptake and further on the protein expression and activity of the insulin signaling pathway. Here we report that UA promotes glucose uptake in adipocytes through the phosphatidylinositol (PI) 3-kinase (PI3K) pathway and enhancing glucose transporter 4 (GLUT4) translocation and expression.

## Materials and Methods

### Chemicals and reagents

Ursolic acid, cytochalasin B, insulin, 3-isobutyl-1-methylxanthine (IBMX), dexamethasone, wortmannin, SB203580, compound C, protease inhibitor and bovine serum albumin (BSA) were purchased from Sigma (St. Louis, MO, USA). High glucose Dulbecco’s modified Eagle’s medium (DMEM) was from Mediatech, Inc. (Cellgro Mediatech, Inc. Manassas, VA). Fetal bovine serum (FBS) was bought from PAA Laboratories (Etobicoke, ON, Canada). Bovine calf serum (BCS) was purchased from Cayman Chemical Company (Ann Arbor, Michigan, USA). The BCA protein assay kit was obtained from Thermo Scientific (San Jose, CA, USA). RIPA lysis buffer was from Millpore (MA, USA). Protein loading buffer was from Bio-Rad (Montreal, QC, Canada). Antibodies against phospho-phosphoinositide-dependent kinase (pPDK), phosphoinositide-dependent kinase (PDK), phospho-protein kinase C (PKC), protein kinase C (PKC), phospho-AS160 (pAS160), AS160, GLUT4, glucose transporter 1 (GLUT1), phospho-phosphoinositide-dependent serine/threonine kinase (pAKT), phosphoinositide-dependent serine/threonine kinase (AKT) and clathrin were from Cell Signaling Technology, Inc. (Beverly, Massachusetts, USA). 2-NBD-glucose was purchased from Invitrogen Life Technologies (Carlsbad, CA, USA). BM chemiluminescence blotting substrate kit was from Roche Diagnosis (Laval, QC, Canada).

### Cell culture

3T3-L1 mouse embryo fibroblasts were obtained from American Type Culture Collection (Rockville, MD) and cultured in DMEM containing 10% BCS until confluent, and were then maintained in the same medium for additional 2 d. The cells were then induced to differentiate according to the method reported previously [13], [24].

### Cell viability

After differentiation, 3T3-L1 adipocytes were cultured in the presence of 2.5 to 50 µM of UA for 24, 48 and 72 h, respectively. At each time point, the cells were treated with MTT assay reagents (1 mg/mL) for 4 h and the resulting formazan was solubilized in 150 µL dimethyl sulfoxide (DMSO) and further diluted 10 times with DMSO. The absorbance was read at 570 nm on a Varioskan Flash spectral scanning multimode plate reader (Thermo Fisher Scientific, Waltham, MA).

### Measurement of glucose uptake

3T3-L1 preadipocytes were cultured in 96-well plates and induced to differentiate for 8 d. The cells were then starved overnight in DMEM with 0.1% BSA and further incubated in Krebs Ringer bicarbonate buffer (110 mM NaCl, 4.4 mM KCl, 1.45 mM KH_2_PO_4_, 1.2 mM MgCl_2_, 2.3 mM CaCl_2_, 4.8 mM NaHCO_3_, 10 mM HEPES and 0.3% BSA) containing 100 µM NBD-glucose, 2.8 mM glucose and different concentrations of UA for 2 h at 37°C in the presence or absence of 1 µg/mL insulin. After 2 h incubation, the reaction was terminated by 20 µM of cytochalasin B. The cells were washed twice with cold Krebs Ringer bicarbonate buffer (4°C), and then 100 uL cold PBS (4°C) were added to each well. The fluorescence intensity was immediately measured at 466/550 nm on a Varioskan Flash spectral scanning multimode plate reader (Thermo Fisher Scientific, Waltham, MA). For the inhibition experiments, the cells were pretreated with PI3K inhibitor wortmannin (1 µM), MAPK inhibitor SB203580 (10 µM), and AMPK inhibitor compound C (2.5 µM), respectively, for 30 min.

### Western blotting

3T3-L1 preadipocytes were grown in 75 cm^2^ flasks and differentiated. After treatment with different concentrations of UA, total protein was extracted using the method described previously [24]. For measuring the translocation of GLUT4, membrane proteins were separated using a membrane protein extraction kit (Abcam, Cambridge, MA) following the manufacturers’ protocol. The protein of GLUT4 was determined using the Western blotting, with clathrin being used as the loading control.

### Immunofluorescence assay

3T3-L1 preadipocytes were cultured in glass-bottomed dishes and differentiated in the induction medium. The differentiated adipocytes were starved in DMEM with 0.1% BSA for 3 h. After pretreatment with wortmannin for 30 min, the cells were treated with 10 µM of UA for 2 h in the presence or absence of 1 µg/mL insulin. They were washed once with ice-cold PBS, fixed for 15 min in 4% paraformaldehyde, permeablized in 0.25% Triton X-100 for 5 min at 4°C, and washed 3 times using ice-cold PBS. After blocking in 10% normal donkey serum at room temperature for 1 h, the cells were washed and incubated at 4°C with anti-GLUT4 antibody overnight. After three washes of 10 min each with PBS, the FITC-conjugated secondary antibody was applied to the samples at room temperature for 1 h. After washing with PBS, images were immediately captured under a Nikon inverted microscope (ECLIPSE TE200).

### Statistical analysis

The statistical analyses were performed using SPSS 13.0 statistical program (version 13.01S; Beijing Stats Data Mining Co. Ltd). The treatment effect was determined using one-way ANOVA and followed by a post-hoc Dunnett's or Bonferroni's multiple comparisons test, where a P value less than 0.05 was considered significant. Data are presented as means ± SD.

## Results

### Effect of UA on fat cell viability

**Figure 1:**
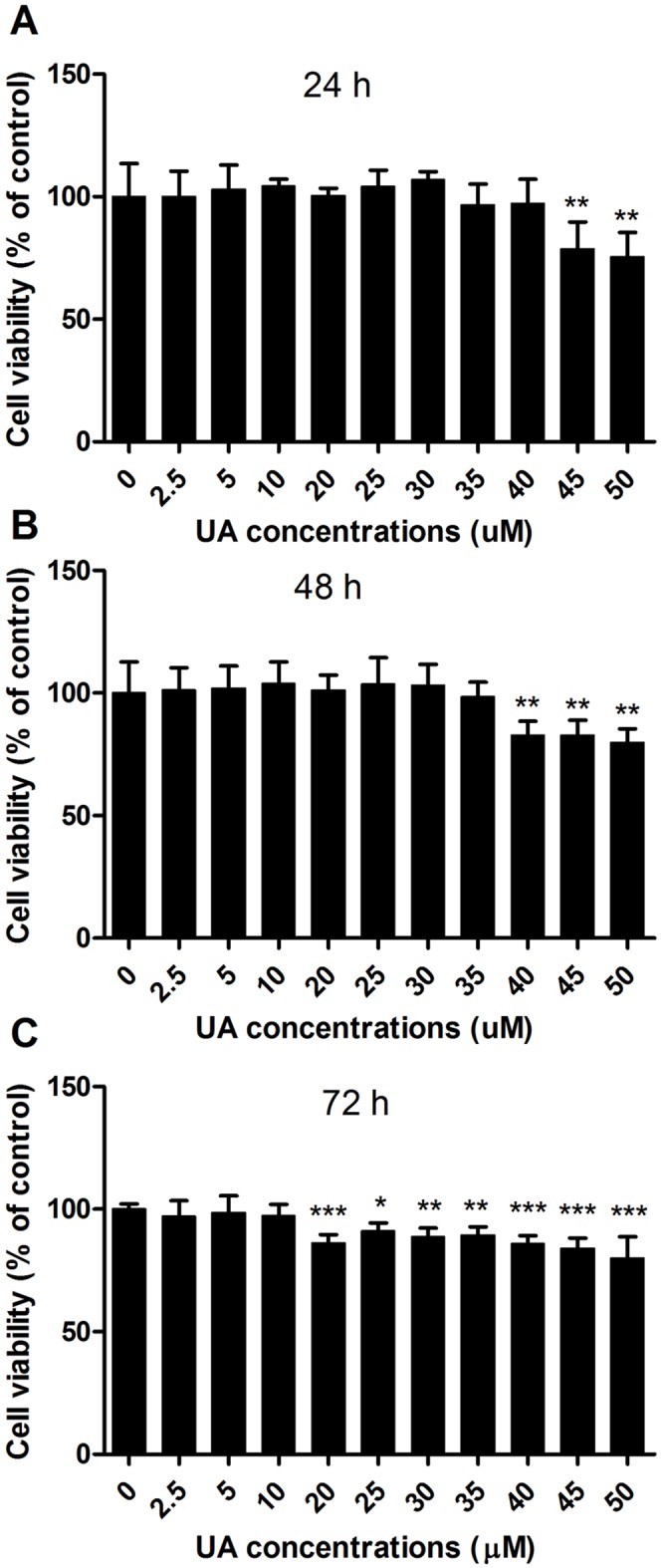
Effect of UA on the viability of 3T3-L1 adipocytes. Mature 3T3-L1 adipocytes were incubated with different concentrations of UA for 24, 48, or 72 h, respectively. The MTT reagents were added to the medium. After 4 h of incubation, the medium was aspirated and 150 µL DMSO was added to each well. The absorbance was measured at 570 nm. Data are expressed as means ± SD (n = 3). *P<0.05, **P<0.001 and ***P<0.001 vs. the control of 0 µM UA.

The MTT assay results revealed that UA at concentrations up to 35 µM did not affect cell viability after 24 or 48 h of incubation (**Figure. 1A, B**), while 20 µM and above being toxic after 72 h of incubation in differentiated 3T3-L1 adipocytes (**Figure 1C**). The concentrations of 20 µM and higher were also toxic in 3T3-L1 preadipocytes [13]. Therefore, a maximal concentration of 10 µM was used in the subsequent experiments.

### UA increases glucose uptake in 3T3-L1 adipocytes

**Figure 2:**
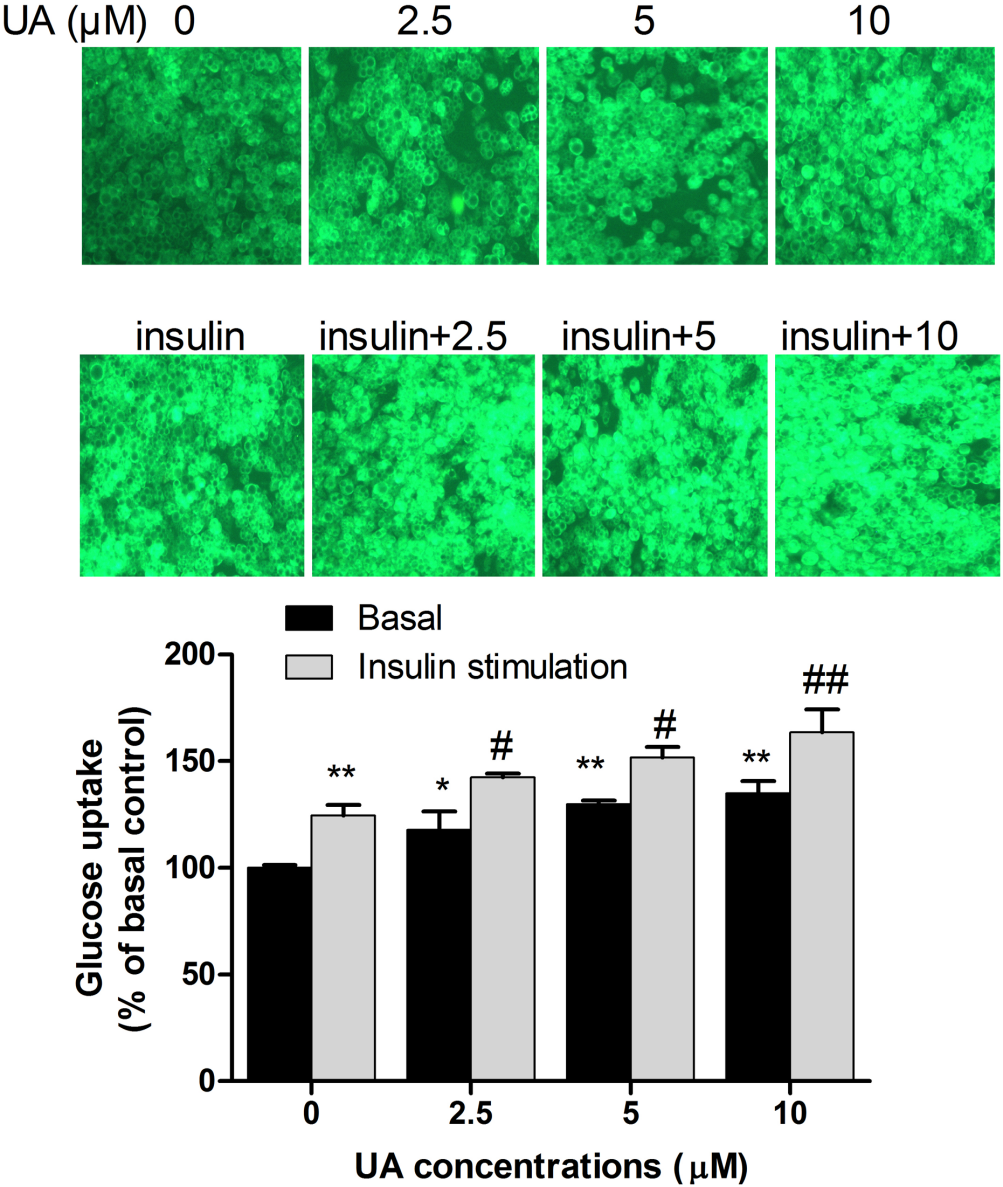
Effect of UA on glucose uptake in 3T3-L1 adipocyte. Mature 3T3-L1 adipocytes were incubated with 2.5 µM, 5 µM, or 10 µM of UA for 2 h and glucose uptake was measured in the presence or absence of 1 µg/mL insulin. The fluorescence intensity of NBD-glucosewas measured at 466/550 nm on a Varioskan Flash spectral scanning multimode plate reader. Data are expressed as means ± SD (n = 3). * P<0.05 and **P<0.01 vs. the control of 0 µM UA; ^#^P<0.05 and ^##^P<0.01 vs. 1 µg/mL of insulin.

To investigate the effect of UA on glucose uptake, differentiated 3T3-L1 adipocytes were cultured in DMEM supplemented with different concentrations of UA for 2 h in the presence or absence of 1 µg/mL of insulin. As shown in **Figure 2**, 2.5, 5, or 10 µM of UA significantly increased glucose uptake by 17%, 29% and 35%, respectively, which was supported by the increase of intracellular fluorescent intensity of NBD-glucose. Glucose uptake was stimulated by insulin at 1 µg/mL and further increased by UA in a dose-dependent manner (**Figure 2**).

### UA stimulates glucose uptake through the PI3K pathway

**Figure 3:**
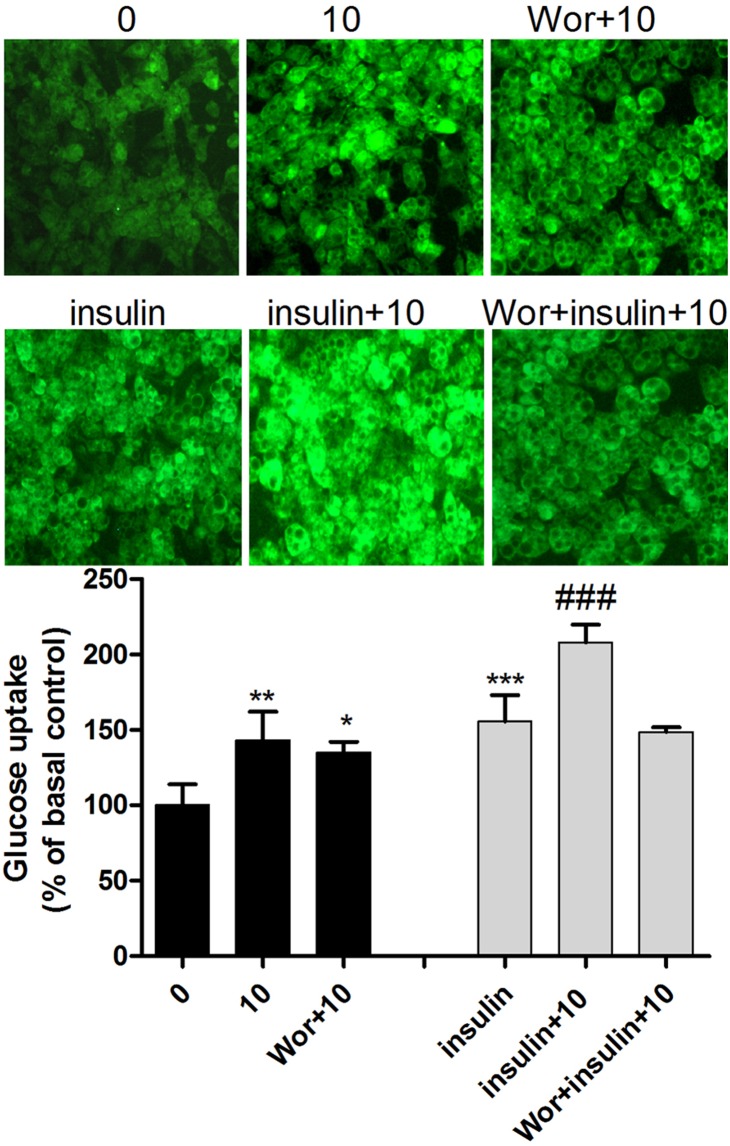
Effect of the PI3K inhibitor wortmannin on glucose uptake in 3T3-L1 adipocyte. Mature 3T3-L1 adipocytes were incubated with 10 µM of UA for 2 h, or pretreated with 1 µM of wortmannin for 30 min before incubation with 10 µM of UA and 1 µM of wortmannin for 2 h. Glucose uptake was measured in the presence or absence of 1 µg/mL insulin. The fluorescence intensity of NBD-glucose was measured at 466/550 nm on a Varioskan Flash spectral scanning multimode plate reader. Data are expressed as means ± SD (n = 3). *P<0.05 and **P<0.01 vs. the control of 0 µM UA; ^#^P<0.05, ^##^P<0.01 and ^###^P<0.001 vs. the insulin control of 1 µg/mL. Wor indicates 1 µM of wortmannin.

To identify the pathway through which UA stimulated glucose uptake, the differentiated 3T3-L1 adipocytes were pretreated with the PI3K, MAPK and AMPK inhibitors, respectively, and then treated with 10 µM of UA in the presence or absence of 1 µg/mL insulin. PI3K inhibitor wortmannin at 1 µM completely blocked the glucose uptake stimulated by insulin, while showing weak effect at the basal level, i.e., in the absence of insulin (**Figure 3**). MAPK inhibitor SB203580 at 10 µM or AMPK inhibitor compound C at 2.5 µM did not have any effect on the UA-stimulated glucose uptake at either the basal or insulin-stimulated status (Fig. S1). These results suggest that the PI3K pathway was responsible for the UA-induced increase of glucose uptake, especially under the conditions of insulin stimulation.

### UA stimulates glucose uptake through regulating protein activities in the PI3K pathway

**Figure 4:**
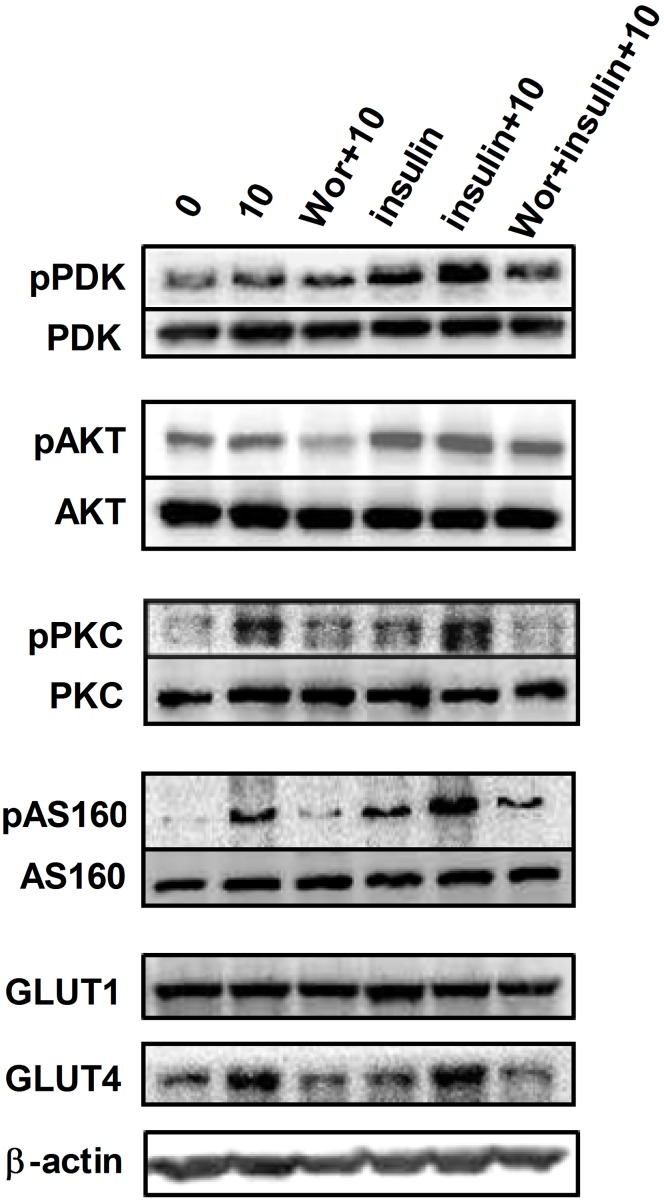
Effect of UA on the activity of PDK, AKT, PKC and AS160, and the expression of GLUT1 and GLUT4. Mature 3T3-L1 cells were treated with 10 µM of UA in the absence or presence of 1 µg/mL insulin for 24 h, or pretreated with 1 µM of wortmannin for 30 min before incubation 10 µM of UA and 1 µM of wortmannin for 24 h. The activity of PDK, AKT, PKC and AS160, and the expression of GLUT1 and GLUT4 were assessed by Western blotting. Data are expressed as means ± SD (n = 3). Wor indicates 1 µM of wortmannin.

To confirm the role of PI3K pathway in upregulating glucose uptake in adipocytes by UA, we further determined the expression or the activity of key proteins of this pathway in the presence or absence of insulin. The activity of PDK and AKT was increased when the cells were treated with 10 µM of UA and more obvious when 1 µg/mL of insulin was applied (**Figure 4**), which was however inhibited by the PI3K inhibitor wortmannin. This observation supported the notion that PI3K pathway was responsible for the enhancing effect of UA on glucose uptake, especially under insulin stimulation in differentiated 3T3-L1 adipocytes. AS160 was a downstream target protein of AKT, and the PKC was a parallel kinase to AKT, which both serve as the effectors facilitating GLUT4 translocation and subsequent glucose uptake [25]. In this study we also observed increases in the activity of AS160 and PKC by UA at 10 µM at either the basal or insulin-stimulated status.

### UA enhances GLUT4 expression and translocation

**Figure 5:**
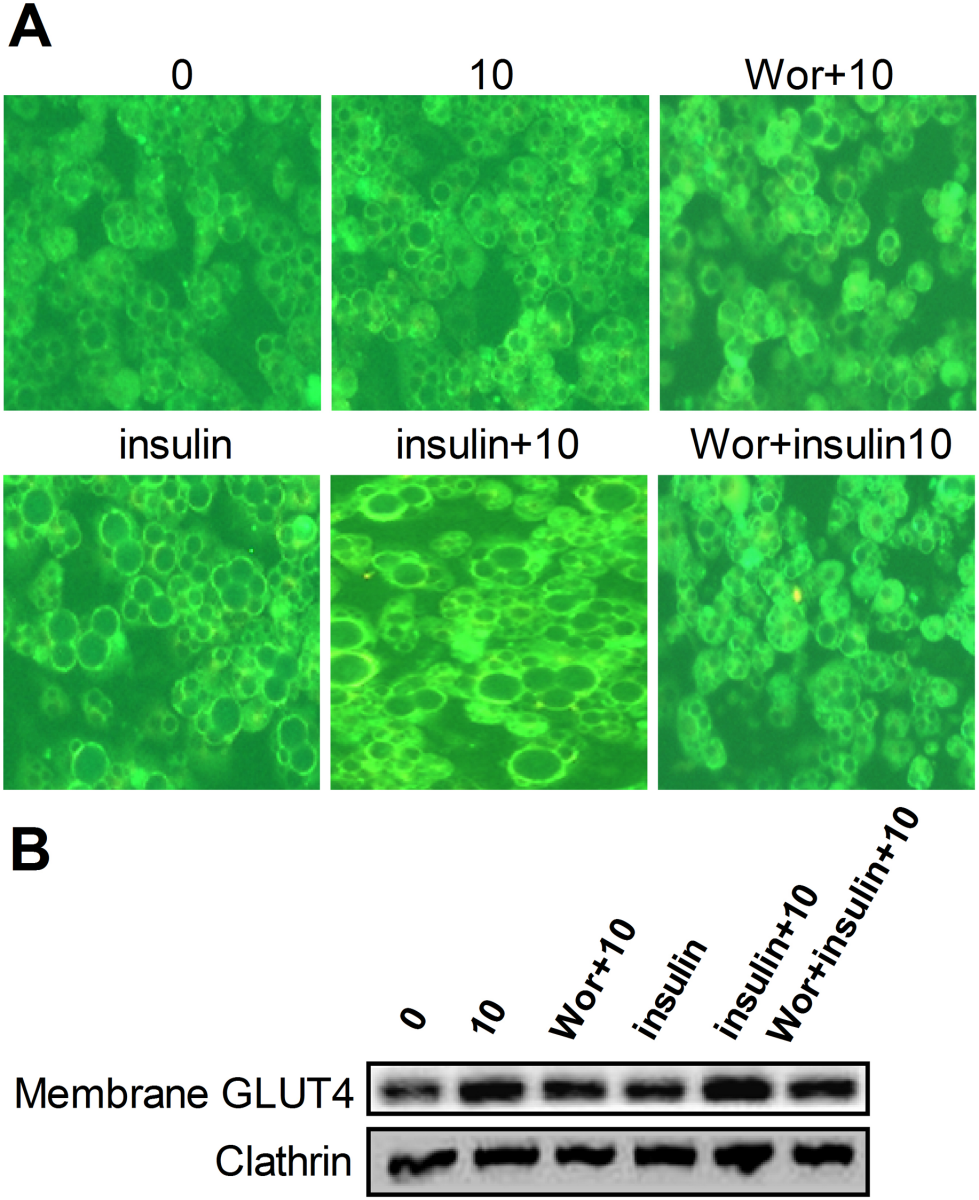
Effect of UA on GLUT4 expression and translocation in 3T3-L1 adipocyte. (A) Mature 3T3-L1 cells were treated with 10 µM of UA in the absence or presence of 1 µg/mL insulin for 24 h, or pretreated with 1 µM of wortmannin for 30 min before incubation with 10 µM of UA and 1 µM of wortmannin. The expression level of GLUT4 was determined by immunofluorescence. (B) Mature 3T3-L1 cells were treated with 10 µM of UA in the absence or presence of 1 µg/mL insulin for 24 h, or pretreated with 1 µM of wortmannin for 30 min and then with 10 µM of UA and 1 µM of wortmannin. Cell membrane was obtained and the expression of GLUT4 was determined with Western blotting (n = 3). Wor indicates 1 µM of wortmannin.

GLUT4 is the key molecule that transports glucose into cells and is critical for glucose uptake by adipose and muscle tissues and thus postprandial glucose clearance from the circulating system. We observed that after 24 h of incubation with 10 µM of UA not only increased the expression of total cellular protein of GLUT4 (**Figure 4**) but also increased its presence on the cell membrane at either the basal or insulin stimulated status (**Figure 5B**). This effect was attenuated by the pretreatment of cells with PI3K inhibitor wortmannin. The result of the immunoflurence assay provided additional support (**Figure 5A**). GLUT1 was reported to function in the process of glucose uptake under a basal status; however, there were not any effects of UA on GLUT1 expression (**Figure 4**). Together, these results imply that the enhancement of GLUT4 expression and translocation was responsible for the observed increase of glucose uptake by UA in 3T3-L1 adipocytes.

## Discussion

Ursolic acid is well known to possess a wide range of biological functions, including anti-inflammatory, anti-oxidative, anti-mutagenic, anti-carcinogenic, hepatoprotective, anti-microbial, anti-atherosclerotic, and anti-hyperlipidemic effects [7], [8]. We have previously demonstrated that UA inhibits adipogenesis through the LKB1/AMPK pathway [13] and promotes lipolysis in adipocytes via the PPARγ signaling [13], and shows an anti-obesity effect *in vivo* [26]. Our recent study showed that UA improved insulin resistance, inflammation and oxidative stress in high-fat diet-induced rats [26]. Obesity is highly related to insulin resistance and T2DM through a mechanism of increased release of fatty acids, glycerol, hormones, pro-inflammatory cytokines and other risk factors from adipose tissue [14]. Adipose insulin resistance contributes to the total insulin resistance and liver complications caused by obesity [27]–[30]. Since UA possesses anti-obesity and anti-insulin resistance effects and has benefits to the adipose tissue fat metabolism, we wondered whether and how UA regulates glucose metabolism in adipocytes. In this study, we demonstrated that UA stimulated glucose uptake in adipocytes through upregulating the PI3K pathway and GLUT4 expression and translocation.

Several studies have demonstrated the hypoglycemic effect of UA [15]–[19]. The reported mechanisms include the inhibition of protein tyrosine phosphatase 1B (PTP1B) [21], increase of liver glycogen synthesis [22], enhancement of pancreatic β-cell function [16] and the activation of skeletal muscle AKT activity [20]. Glucose homeostasis is determined by glucose production and utilization in the insulin-sensitive organs and tissues, including muscle, liver and adipose tissue. Glucose uptake in adipose tissue plays a critical role in the body glucose control [23], which is demonstrated by the selective depletion of GLUT4 in adipose tissues of mice [31]. We observed that UA stimulated glucose uptake in adipocytes in a dose-dependent manner at either the basal or insulin-stimulated status. Wortmannin is a PI3K inhibitor, when it was added in the culture medium of 3T3-L1 adipocytes, the effect of UA on glucose uptake was almost completely abolished. PDK is the downstream target of the PI3K and functions as an activator of AKT [25]. UA activated the PDK and AKT in the presence of insulin and again this effect was attenuated by wortmannin. PKC is a parallel kinase to AKT [32] and AS160 is the substrate of AKT [25]. They are all involved the regulation of GLUT4 translocation. Interestingly, the UA alone or together with insulin increased the activities of PKC and AS160, both are important effectors in modulating glucose uptake [25]. These effects of UA were suppressed by the PI3K inhibitor. The findings suggest that the PI3K pathway is responsible for the UA-stimulated glucose uptake in adipocytes.

Although the results of the present study suggested that UA enhanced glucose uptake through the PI3K pathway, we could not rule out other pathways that may also contribute to the increased glucose uptake in adipose tissue. MAPK has been shown to modulate glucose uptake stimulated by insulin or other factors in adipocytes and muscle cells [33], [34]. AMPK is reported to be a key regulator of glucose transport in 3T3-L1 adipocytes [35]. We demonstrated previously that UA inhibited adipogenesis in 3T3-L1 adipocytes through stimulating the AMPK activity [13], suggesting a possible role of AMPK in the UA-enhanced glucose uptake. To understand the role of these molecules in UA-regulated glucose uptake, we further investigated the effect of the kinase inhibitors on the UA-induced increase of glucose uptake in adipocytes. Surprisingly, AMPK inhibitor (compound C) or MAPK inhibitor (SB203580) did not block the stimulatory effect of UA on glucose uptake, indicating that the regulation of UA on glucose uptake in adipocytes was independent of the MAPK and AMPK pathways.

Glucose uptake in adipocytes is regulated by glucose transporters, GLUT1 and GLUT4 [36], [37]. Of them, GLUT1 is essential for basal glucose transport in various tissues, whereas GLUT4 is selectively expressed in the insulin-responsive tissues, such as adipose and skeletal muscle cells. UA did not show any effect on GLUT1 expression but significantly upregulated GLUT4 levels in adipocytes. When blood glucose level is elevated, insulin is released into the blood stream and triggers insulin signaling. The GLUT4 moves from cytoplasm to the cell membrane where it transports glucose across the membrane into the cells. Thus, the total expression and translocation of GLUT4 is critical in glucose transport and uptake, and subsequently glucose disposal and clearance from the circulation. Both the protein expression and immonofluorescence assay results revealed that UA upregulated GLUT4 expression and translocation, in good agreement with the increased activity of AS160, which is a key regulator of GLUT4 trafficking in 3T3-L1 adipocytes [38], [39]. This notion was further supported by the effect on PKC that regulates GLUT4 distribution in 3T3-L1 adipocytes [40]. We did not determine the effect of UA on the activity of insulin receptor and insulin receptor substrate, which are important proteins at the upstream of the PI3K pathway. However, UA has been reported to increase insulin receptor phosphorylation in the presence of insulin [18]. It should be pointed out that UA increases the phosphorylation and activity of AMPK and the expression of CPT1, thus inhibits fatty acid synthesis while enhancing fat oxidation [13]. As a result, UA stimulated glucose uptake but did not promote the synthesis and accumulation of fat in adipocytes.

In conclusion, UA increased glucose uptake in 3T3-L1 adipocytes by activating the PI3K pathway and GLUT4 translocation. The results provide additional information regarding the mechanism of action of UA for its anti-diabetic effect.

## Supporting Information

Figure S1 **Effect of the AMPK inhibitor compound C or the MAPK inhibitor SB203580 on glucose uptake in 3T3-L1 adipocyte.** Mature 3T3-L1 adipocytes were incubated with 10 µM of UA for 2 h, or pretreated with 2.5 µM of coumpound C or pretreated with 10 µM of SB203580 for 30 min before incubation with 10 µM of UA in the presence of indicated concentrations of inhibitors for 2 h. Glucose uptake was measured in the presence or absence of 1 µg/mL insulin. The fluorescence intensity of NBD-glucose was measured at 466/550 nm on a Varioskan Flash spectral scanning multimode plate reader. Data are expressed as means ± SD (n = 3). *P<0.05 and **P<0.01 vs. the control of 0 µM UA; CC indicates 2.5 µM of coumpound C and SB indicates 10 µM of SB203580.
(TIF)
