# Supplementary figures and images for: Ursolic Acid Increases Glucose Uptake through the PI3K Signaling Pathway in Adipocytes
Source: PLoS One. 2014 Oct 20;9(10):e110711. doi: 10.1371/journal.pone.0110711 (PMC4203820; doi:10.1371/journal.pone.0110711)

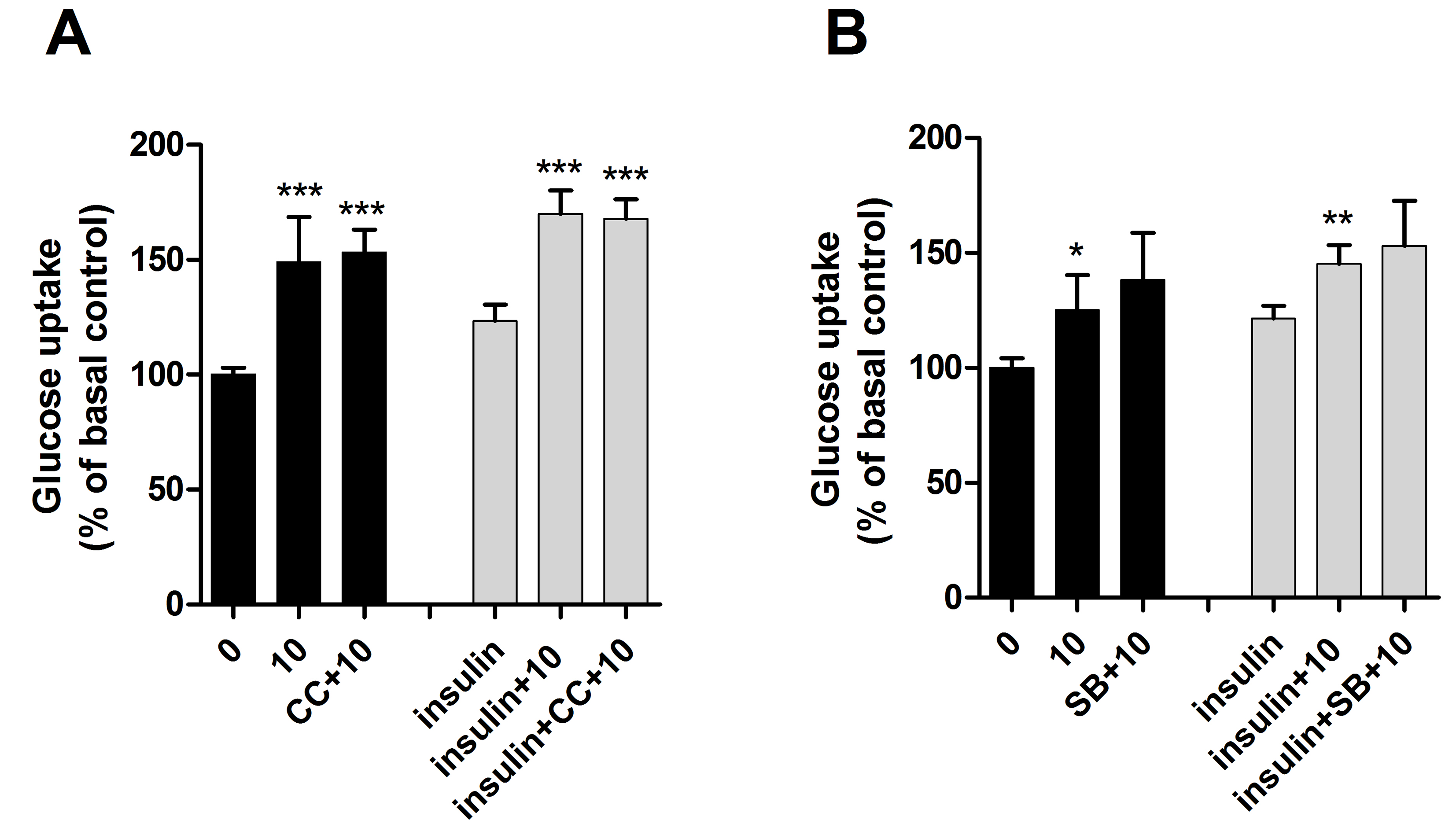

Supplement: Figure S1 — Effect of the AMPK inhibitor compound C or the MAPK inhibitor SB203580 on glucose uptake in 3T3-L1 adipocyte. Mature 3T3-L1 adipocytes were incubated with 10 µM of UA for 2 h, or pretreated with 2.5 µM of coumpound C or pretreated with 10 µM of SB203580 for 30 min before incubation with 10 µM of UA in the presence of indicated concentrations of inhibitors for 2 h. Glucose uptake was measured in the presence or absence of 1 µg/mL insulin. The fluorescence intensity of NBD-glucose was measured at 466/550 nm on a Varioskan Flash spectral scanning multimode plate reader. Data are expressed as means ± SD (n = 3). *P<0.05 and **P<0.01 vs. the control of 0 µM UA; CC indicates 2.5 µM of coumpound C and SB indicates 10 µM of SB203580. (TIF) [file pone.0110711.s001.tif]
